# Association of epidermal growth factor receptor and K-Ras mutations with smoking history in non-small cell lung cancer patients

**DOI:** 10.3892/etm.2012.829

**Published:** 2012-11-23

**Authors:** ONUR BAYKARA, MERVE TANSARIKAYA, AHMET DEMIRKAYA, KAMIL KAYNAK, SERHAN TANJU, ALPER TOKER, NUR BUYRU

**Affiliations:** 1 Departments of Medical Biology, Cerrahpasa Medical Faculty, Istanbul University, Kocamustafapasa, Istanbul 34098;; 2 Chest Diseases, Cerrahpasa Medical Faculty, Istanbul University, Kocamustafapasa, Istanbul 34098;; 3 Department of Chest Diseases, Istanbul Medical Faculty, Istanbul University, Capa, Istanbul 34093, Turkey

**Keywords:** non-small cell lung cancer, epidermal growth factor receptor, K-Ras, smoking

## Abstract

Lung cancer, a major health problem affecting the epithelial lining of the lower respiratory tract, is considered to be one of the deadliest types of cancer in males and females and it is well-known that smoking is the chief cause of lung cancer. In addition to smoking and environmental factors, genetic susceptibility may also contribute to the development of lung cancer. Previous studies have shown that certain non-small cell lung cancer (NSCLC) patients harbor gain-of-function mutations in the epidermal growth factor receptor gene (EGFR). Phosphorylated EGFR triggers the activation of intracellular signal transduction pathways, including the RAS-MAPK, PI3K-Akt and STAT pathways. However, K-Ras gene point mutations in codons 12, 13 or 61 cause the inactivation of GTPase activity which results in overstimulation of cellular growth and gives rise to neoplastic development. Our aim was to investigate the presence and association of EGFR and K-Ras mutations in 50 primary NSCLC patients with a smoking history by using real-time PCR and sequencing. EGFR mutations were detected in four patients (8%). Two of these mutations were L858R mutations and the remaining two were deletion mutations spanning between codons 746 and 750. The L858R mutation was significantly associated with smoking status (P=0.003). K-Ras codon 12 and 61 mutations were also observed in four patients. However, no association was observed between K-Ras mutations and the tumor staging, gender, histology and smoking status of the patients.

## Introduction

Lung cancer, which involves malignant proliferation of the epithelial lining of the lower respiratory tract, is the most common cause of cancer mortality in males and is second only to breast cancer in females (1–3). Smoking is considered to be one of the principal causes of lung cancer. However, as only a subgroup of smokers ever develop lung cancer, it has been suggested that genetic susceptibility may significantly contribute to the risk of the disease (4,5). Therefore, various genetic factors, including mutations or overexpression of oncogenes and functional inactivation of tumor suppressor genes, have been implicated in the development of lung cancer (6,7).

There have been a number of studies showing that gain of function mutations in the epidermal growth factor receptor (EGFR) gene may cause non-small cell lung cancer (NSCLC) (8,9). EGFR is a 170-kDa transmembrane tyrosine kinase receptor that is present in the majority of epithelial tissues and is important in cell growth and function. The EGFR signaling system is activated by three sequential steps. First, specific ligands bind to the extracellular domain of EGFR, resulting in a conformational change. Second, this structural change allows the receptor to form a dimer with another ligand-bound EGFR. This dimerization event then causes autophosphorylation of tyrosine kinase within the intracellular domain of the receptors, leading to the activation of signal transduction pathways. EGFR tyrosine phosphorylation triggers several signaling cascades, including the RAS-MAPK, PI3K-Akt and STAT pathways.

The K-Ras gene encodes a 21-kDa G-protein with GTPase activity that functions downstream of EGFR-induced cell signaling. K-Ras is the most commonly mutated oncogene in lung cancer with a mutation frequency of 3–35% (10–12). The hallmark of RAS function is a switch between the inactive GDP (guanosine diphosphate-bound) state and active GTP (guanosine triphosphate-bound) states. Point mutations in codons 12, 13 or 61 appear to inhibit the GTPase activity of the ras protein, resulting in constitutive stimulation of autonomous growth which contributes to neoplastic development (13).

Given the importance of the EGFR in tumorogenesis and disease progression, this receptor has become a relevant and promising target for anticancer therapies. However, point mutations in codons 12 and 13 of the K-Ras oncogene may interfere with otherwise intact EGFR signaling, leading to a lack of response to EGFR inhibitors, and are consequently correlated with poor responses to EGFR-targeted therapies (14–16). Therefore, knowledge of the EGFR and K-Ras mutation status of a patient’s tumor is likely to provide a potential strategy for selecting those patients who are likely to benefit from EGFR-targeted therapies. To the best of our knowledge, there are only a few studies in the literature investigating the EGFR and K-Ras mutations in NSCLC tumor samples simultaneously (10–18). This is also the first report investigating these mutations in Turkish NSCLC patients.

## Materials and methods

### Sample analysis

The tumor and corresponding normal lung tissues and blood samples were obtained from 50 patients undergoing surgery at Istanbul University Cerrahpasa Medical Faculty, Department of Chest Surgery and Istanbul University Istanbul Medical Faculty, Department of Chest Surgery (Turkey). DNA was isolated by digestion of the tumor and corresponding normal tissue samples using a DNA isolation kit (Tissue DNA Isolation kit, Macherey Nagel, Düren, Germany) according to the manufacturer’s instructions and the DNA was kept at −80°C until used. The TheraScreen mutation kit (DxS Limited, Manchester, UK) was used to analyze a total of 29 mutations in the EGFR gene and seven mutations in the K-Ras gene by real-time PCR. EGFR and K-Ras gene mutation analysis was performed by sequencing analysis for confirmation, following the determination of the mutations by real-time PCR. For this purpose, exons 18–21 of the EGFR gene and two exons of the K-Ras gene were sequenced using appropriate primers.

### Ethics

An explanatory statement concerning the study and the procedures was made to all patients. The statement was a regulated ethics statement prepared by The Ethics Committee of Istanbul University Cerrahpasa Medical Faculty. The committee approved the study and written informed consent was obtained from all patients.

## Results

### Patient characteristics

The aim of the present study was to investigate the presence and correlation of EGFR and K-Ras mutations and their association with smoking history in a series of 50 primary NSCLC tumors. Out of 50 patients diagnosed as having NSCLC, 21 (42%) had squamous cell carcinoma, 20 (40%) had adenocarcinoma and 9 (18%) had tumors of various histologies. The patients comprised 44 (88%) males and 6 (12%) females. The majority of the patients (52%) had stage I disease and were smokers (90%).

### EGFR mutations

Genetic alterations in the EGFR gene were detected in four (8%) patients. Two of these were L858R mutations, while the remaining two were deletion mutations spanning codons 746 to 750. Out of four tumor samples with EGFR mutations, three were adenocarcinomas and the remaining one was NSCLC with unidentified histology. The two patients carrying EGFR L858R mutations were smokers. One of the patients smoked 30 packs/year and the other smoked 50 packs/year (20 cigarettes/pack). When the correlation between the smoking status and EGFR mutation type was analyzed, a statistically significant association was observed between L858R mutation and smoking status (P=0.003; Table I), but there was no correlation with stage, gender and histological type (Table I).

### K-Ras mutations

K-Ras mutations were observed in four (8%) patients. One (25%) of these was a codon 61 (Gln61His; CAA>CAT) mutation, two were codon 12 (Gly12Cys; GGT>TGT) mutations and the remaining one was another codon 12 (Gly12Val; GGT>GTT) substitution. Three of the mutated samples were adenocarcinomas and one was a histologically large cell carcinoma. We identified no associations between the mutations in the K-Ras gene and stage, gender, histologic type and smoking status (Table I).

## Discussion

NSCLC is the most common form of lung cancer and results from the accumulation of multiple genetic abnormalities. Extensive research on the EGFR molecule has revealed its oncogenic role, particularly in NSCLC and colorectal carcinoma (8,9,19,20). These advances have also led to the development of new therapeutic agents targeting EGFR. It is known that EGFR is overexpressed in the majority of cases of NSCLC (10,17,21,22) and that mutations in the K-Ras gene are also frequent (23,24). The EGFR mutation frequency in lung tumors has been reported to be between 9.8 and 44% in various populations (8,9,20,25,26). To the best of our knowledge there is no study in the literature investigating the co-existence of EGFR and K-Ras mutations in Turkish patients with NSCLC. In the present study, EGFR mutations were observed in 8% of the patients. This was lower than previously reported frequencies for other populations (8,9,20,25,26). This finding may be associated with the smoking status of the patients. A number of investigators have suggested that the EGFR mutation rate decreases when the smoking dose increases (27). In the population of the present study, 90% of the patients were smokers and 51% had been smoking >30 packs/year. The results of the present study are in accordance with the EGFR mutation frequencies observed when investigating the association between smoking status and EGFR mutations in NSCLC patients. The mutation frequency at exons 18–21 of the EGFR gene identified in previous studies has ranged from 9.8 to 17% in the smoking group (27–30). Lee *et al*(30) also reported that the frequency of the EGFR mutations was significantly lower in patients who smoked >25 packs/year or who had quit smoking <10 years ago. In the majority of studies, EGFR mutations have been correlated with patient characteristics such as histological type, gender or ethnic origin (9,25,31). However, there are no reports in the literature investigating an association between the EGFR mutation and the stage of the tumor. Statistical analysis of the present results, revealed a significant association between the EGFR mutation and the stage of the tumor. This result indicates that EGFR mutation is not an early event in NSCLC but occurs frequently at the late stage of the disease. However, a statistically significant association was observed between the smoking status and the L858R mutation. The L858R mutation was more frequent in smokers than the del746–750 deletion in the EGFR gene.

In previous studies, K-Ras mutations have frequently been investigated and associated with colorectal carcinoma (32–34). Point mutations in the K-Ras gene have also been associated with tobacco smoking in NSCLC (23,35). In the present study, a correlation was not observed between the smoking dose and K-Ras mutation. Studies investigating the presence of the K-Ras mutation and its co-existance with the EGFR mutations in NSCLC are rare and inconsistent (10–12,18). In the present study, EGFR and K-Ras mutations were not observed concurrently in the same patient, supporting the theory that K-Ras and EGFR mutations are mutually exclusive (8,36). In the present study group, the K-Ras mutation frequency was the same as the EGFR mutation frequency (8%). These results are in accordance with the findings of previously reported studies (10–12,16,18) but contrast with the data of Schmid *et al*(37) who reported concomitant K-Ras and EGFR mutations in 2% of patients. The presence of somatic mutations in the K-Ras oncogene has been considered to be a marker of the lack of response to EGFR-targeted therapies (10,18,38–41). Results of a meta-analysis also indicated that 20% of NSCLC patients may be non-responsive to EGFR-targeted therapies due to an underlying K-Ras mutation (16).

In conclusion, the results of the present study suggest that, in addition to an inverse correlation between smoking history and the presence of EGFR mutations, the type of EGFR mutation is also correlated with smoking status and smoking dose. In view of these findings, we propose that the main causal factor of NSCLC in Turkey is smoking rather than the activation of the oncogenic EGFR pathway.

## Figures and Tables

**Table I.** Distribution of clinical parameters in NSCLC patients.

Factor	n	K-Ras mutation n (%)	Pearson’s P-value	EGFR mutation n (%)	Pearson’s P-value
Gender					
Female	6	2 (33.3)	0.013	2 (33.3)	0.065
Male	44	2 (4.5)		2 (4.5)	
Histological type					
Squamous cell	21	-		-	0.073
Adenocarcinoma cell	20	3 (15)		3 (15)	
Large cell	5	1 (20)	0.81	-	
Mixed	2	-		-	
Undetermined	2	-		1 (50)	
Stage					
I	26	3 (11.5)		-	0.002
II	6	-		-	
III	8	-	0.82	1 (12.5)	
IV	1	-		1 (100)	
Undetermined	9	1 (11.1)		2 (22.2)	
Smoking status					
Smoker	45	3 (6.6)	0.024	2 (4.4)	0.005
Non-smoker	5	1 (20)		2 (40)	0.003

NSCLC, non-small cell lung cancer; EGFR, epidermal growth factor receptor.
